# Leveraging the microbiome to combat antibiotic resistant gynecological infections

**DOI:** 10.1038/s44259-025-00106-2

**Published:** 2025-04-23

**Authors:** Tanya Kumar, Aryak Rekhi, Yumie Lee, Julielam Tran, Arlene Grace D. Nagtalon, Sidhant Rohatgi, Erika L. Cyphert

**Affiliations:** 1 University of California San Diego, Shu Chien-Gene Lay Department of Bioengineering, La Jolla, CA USA; 2 Medical Scientist Training Program, University of California San Diego, La Jolla, CA USA

**Keywords:** Metabolomics, Proteomics, Sequencing, Infectious diseases

## Abstract

The vaginal resistome can be considered a collection of the resistant determinants in the vaginal microbiome. Here we review the vaginal resistome including the microbes and resistant genes harbored in common gynecological infections, vaginal microbes that participate in horizontal gene transfer, host factors that contribute to the resistome, and common therapies. Finally, we provide perspective on technologies that can be leveraged to study the vaginal resistome and remaining challenges.

## Introduction to the vaginal resistome

The emergence of multidrug resistant pathogens due to the decreasing efficacy of existing antibiotics is expected to contribute to 10 million deaths per year by 2050^1^. While many highly resistant pathogens in the gut, such as vancomycin-resistant *Enterococci* have been well studied with various attempts to overcome it underway^2^, the resistance profile of pathogens within the vaginal microbiome largely remains elusive. Compared to the gut microbiome, the vaginal microbiome is a relatively understudied and low biomass system (10^10^–10^11^ CFU) that is gaining recognition as a key player in healthcare for billions of people globally^3–5^. The presence of resistance phenotypes in the vaginal microbiome poses a major threat to human health, with some infections leading to infertility, widespread scarring in the genital tract, miscarriage and preterm birth, and an increased risk of co-infections with human immunodeficiency virus (HIV)^6–8^. As such, the vaginal microbiome is not just a women’s health topic, but one that has lasting impacts on the global population. Inspired by the term “antibiotic resistome” which is defined as “resistance determinants present in the soil,” in this work we define the “vaginal resistome” as the resistant determinants present in the vagina that includes antimicrobial resistance genes, metabolites, and physical characteristics of the microenvironment that lead to a resistant phenotype^9,10^. In this section, we discuss various contributions to the vaginal resistome, both on the host and pathogen side, and mechanisms of transmission. As a note, use of the term “female” refers to those with a female reproductive tract, although the majority of studies cited were performed with cis-gendered female participants.

### Microbes contributing to the vaginal resistome

A number of vaginal microbes harbor antimicrobial resistance genes and contribute to the vaginal resistome. Here we focus on several common resistant gynecological infections including bacterial vaginosis (BV), vulvovaginal candidiasis, *Neisseria gonorrhoeae, Chlamydia trachomatis*, and Group B Streptococcus (caused by *Streptococcus agalactiae*) and the resistance genes that they carry.

BV is the most common global cause of vaginitis in women of reproductive age and is a non-sexually transmitted infection (STI) biofilm-based disease associated with an altered vaginal microbiome^11^. While it is difficult to make a clear distinction between the composition of a “healthy” and “unhealthy” microbiome, there is consensus that a microbiome dominated by lactic acid producing microbes (mainly of the genus *Lactobacillus*), help constitute an acidic and non-diseased state^8^. Beyond production of lactic acid, *Lactobacillus spp*. can help to ward off pathogen growth by secreting bacteriocidal and bacteriostatic compounds^12^. BV is classically associated with a decreased abundance of *Lactobacilli* and is characterized by an overgrowth of anaerobes (some natively occurring) including *G. vaginalis*, *Fannyhessa (Atopobium) vaginae, P. bivia, Mobiluncus mulieris*, and *Sneathia*, among others often forming a dense polymicrobial biofilm^13–17^. However, it is important to note that a vaginal microbiota dominated by anaerobes does not necessarily lead to clinical presentation or asymptomatic BV. While *G. vaginalis* is a major component of BV biofilms, other *Gardnerella* species (*G. leopoldii*, *G. piotii*, and *G. swidsinskii*), *F. vaginae*, and *P. bivia* can comprise the polymicrobial biofilm^17–19^. Notably, when treated with antibiotics, BV has an estimated recurrence rate from 30–80%, depending on the patient population and treatment modality^20,21^. BV biofilms are often resistant to antibiotics and can recur due to the existence of persister cells that have decreased metabolic activity and the physical barrier the biofilms create blocking antibiotics from accessing microbes within the extracellular polymeric substances, among other mechanisms^15,22,23^. Furthermore, there is an increased rate of mutation and horizontal gene transfer amongst microbes in biofilms relative to microbes in their planktonic state, which are key methods of establishing and spreading antimicrobial resistant genes, as outlined in the section “Horizontal gene transfer and spread of the vaginal resistome”^24^. *G. vaginalis* has been shown to carry resistance genes to tetracycline, fluoroquinolone, and macrolide/lincosamide/streptogramin (MLS)^25^. One of the earliest detections of resistance in vaginal microbes was of *tet(M)* (coding for tetracycline resistance) in *G. vaginalis* in the 1990s^26^.

Vulvovaginal candidiasis (commonly known as a “yeast infection”) is canonically caused by the dimorphic fungus *Candida spp*. (*C. albicans, C. glabrata, C. tropicalis, C. parapsilosis*) and is responsible for 20–25% of vaginitis cases^27,28^. Similar to BV pathogens, *Candida spp*. can form biofilms and cause recurrent infections and may naturally occur at low levels in the vaginal microbiome^29^. Formation of a biofilm has been shown to significantly increase antimicrobial resistance, altering the resistome^30^. *Candida spp*. has shown resistance to antifungal treatments including azoles (fluconazole, itraconazole, voriconazole) and amphotericin B^31,32^. Resistance in *Candida spp*. has been linked with changes in gene expression including *EGR11, CDR1, CDR2*, and *MDR1* which encode the target enzyme and efflux pumps, respectively^32^. Further, cross-resistance has been detected amongst azoles due to similar resistance mechanisms including fluconazole, ketoconazole, clotrimazole, and itraconazole^32^.

*N. gonorrhea*, *Chlamydia trachomatis*, and *Treponema pallidum* are common bacterial STIs that can carry antimicrobial resistance genes and are typically not a major component of the vaginal microbiome. Antimicrobial resistance in *N. gonorrhea* is particularly concerning and is considered a global health issue^33^. *N. gonorrhea* can harbor antimicrobial resistance plasmid-derived genes related to efflux pumps and porins including *bla*_*TEM*_*, tet(M), penA, ponA, mtrR*, and *porB1*^34–36^. *Chlamydia trachomatis* can develop resistance to several classes of antibiotics including macrolides, rifamycins, tetracyclines, fluoroquinolones, aminoglycosides, and fosfomycin^37^. Antimicrobial resistance genes associated with *Chlamydia trachomatis* include *rplD* (macrolide), *rplV* (macrolide), *rpoB* (rifamycin and tetracycline), *gyrA* (fluoroquinolone), *parC* (fluoroquinolone)*, ygeD* (fluoroquinolone), *murA* (Fosfomycin), *secY*, and *tet(C)* (tetracycline)^37^. Both *N. gonorrhea* and *Chlamydia trachomatis* are also capable of forming polymicrobial biofilms, further decreasing their susceptibility to antibiotics^38,39^. *T. pallidum* has been shown to develop resistance to macrolides through a mutation to the 23S rRNA gene^40^.

While typically not constituents of the vaginal microbiome, cervicovaginal infections involving *Escherichia coli* and methicillin-resistant *Staphylococcus aureus* (MRSA) pathogens can also colonize the vaginal microbiota^41–44^. Cervicovaginal *E. coli* can harbor resistance to a variety of antibiotic classes including aminoglycoside, tetracycline, beta-lactam, and sulfamethoxazole (*aac(3)II, TEM, dfrA1, sul1, qnrA*)^42–44^. MRSA cervicovaginal infections have been associated with resistance to erythromycin, doxycycline, and mupirocin (*ermA, ermC*)^45^. Additionally, *Streptococcus agalactiae* (Group B Streptococcus) can reside in the vaginal microbiome of pregnant individuals and cause serious complications when spread to newborns^45–47^. Group B Streptococcus has shown resistance to macrolides, lincosamides, and quinolones (*ermA, ermB, mefA/B*)^46–48^.

Not only have BV microbes acquired resistance genes, but commensal bacteria as well have demonstrated a reservoir for resistance. Specifically, *Lactobacillus spp*. have shown resistance to metronidazole, sulfamethoxazole, and kanamycin and can harbor *tet(M)*, *tet(K)*, and *ermB* resistance genes^49^. Additionally, Jeters et al. detected *tet(M)*, *tet(W)*, *tet(Q)*, *ermB*, and *ermF* in the vaginal resistome of primates that were not exposed to antibiotics^50^. Zhang et al. also made the troubling discovery of colistin resistance genes (*mcr-1, mcr-2, mcr-3, mcr-4, mcr-5*) in the vaginal microbiome in a cohort of women being evaluated for infertility^51^. Collectively, suggesting that even in the absence of antibiotic use, the vaginal microbiome can harbor antimicrobial resistance genes. A summary of antimicrobial resistance genes associated with vaginal microbes is highlighted in Table 1.

**Table 1:** Relationships between antimicrobial resistance genes and growth of resistant microbes in the vaginal microbiome

Antimicrobial resistance gene	Class of resistance	Associated resistant microbes	Key technique(s)	Reference
*catI* *ErmF* *Mel* *CfxA2* *tet(32)* *Tet-res-rpp* *IsaC* *ileS* *ErmA* *tet(W)* *tet(O)* *rpoB mutants* *tet(M)* *tet(Q)*	Phenicol MLS MLS Beta-lactam Tetracycline -- -- Mupirocin-like MLS Tetracycline Tetracycline Rifamycin Tetracycline Tetracycline	*Prevotella*, *Gardnerella*, *Lactobacillus*, *Mobiluncus*, *Sneathia*, *Megasphaera*, *Fannyhessa*, *Bifidobacterium*, *Porphyromonas*	Shotgun metagenomics	Roachford et al. ^81^.
*tet(M)* *tet(W)* *tet(Q)* *ermB* *ermF*	Tetracycline Tetracycline Tetracycline MLS MLS	*Firmicutes*, *Bacteroides*	PCR resistance genes	Jeters et al. ^50^.
*tet(M)* *tet(K)* *ermB*	Tetracycline Tetracycline MLS	*Lactobacillus*	PCR resistance genes	Stsepetova et al. ^49^.
*blaTEM*	Beta-lactam	*N. gonorrhea*	PCR resistance genes	Muhammad et al. ^34^.
*tet(M)*	Tetracycline	*N. gonorrhea*	PCR resistance genes	Młynarczyk-Bonikowska et al. ^35^.
*penA* *ponA* *mtrR* *porB1*	Beta-lactam Beta-lactam MLS --	*N. gonorrhea*	--	Osawa et al. ^36^.
*rplD* *rplV* *rpoB* *gyrA* *parC* *ygeD* *murA* *secY* *tet(C)*	MLS MLS Rifamycin Fluoroquinolone Fluoroquinolone Fluoroquinolone Fosfomycin -- Tetracycline	*Chlamydia*	PCR resistance genes	Benamri et al. ^37^.
*tet(M)* *tet(L)*	Tetracycline Tetracycline	*E. faecalis*, *S. anginosus*	PCR resistance genes	Sirichoat et al. ^41^.
*aac(3)II* *TEM* *dfrA1* *sul1* *qnrA*	Aminoglycoside Beta-lactam Diaminopyrimidine Sulfonamide Fluoroquinolone	*E. coli*	PCR resistance genes	Monroy-Pérez et al. ^42^.
*ermA* *ermC*	MLS MLS	Methicillin-resistant *S. aureus* (MRSA)	PCR resistance genes	Chadwick et al. ^45^.
*ermA* *ermB* *mefA*	MLS MLS MLS	*S. agalactiae* (Group B strep)	PCR resistance genes	Domelier et al. ^47^.
*mcr-1* *mcr-2* *mcr-3* *mcr-4* *mcr-5*	Peptide Peptide Peptide Peptide Peptide	*Enterobacteriaceae*	PCR resistance genes	Zhang et al. ^51^.

### Horizontal gene transfer and spread of the vaginal resistome

Horizontal gene transfer is the primary method by which bacteria exchange resistance genes after they are established via random mutation^52–55^. Transfer of multiple genes located close together on conjugative plasmids can result in acquisition of antimicrobial resistance genes to antibiotics that an individual has never been exposed to^56^. Horizontal gene transfer can occur via multiple mechanisms including conjugation, transduction, and transformation and it is challenging to map mobile genetic elements to their hosts in biofilms and microbial consortia^52–55^. Recent advances in single-molecule DNA and multiplexed ribosomal RNA fluorescence in situ hybridization (FISH) have enabled mapping and visualization of taxa and mobile genetic elements in human plaque biofilms, however, there have yet to be analogous studies on biofilms in the vaginal microbiome^57,58^. Alternatively, shotgun metagenomic sequencing techniques, such as high-throughput chromatic conformation capture (Hi-C), have enabled chromosomal and extrachromosomal resistance genes and other mobile genetic elements to be traced to their microbial origin^59–62^. However, at this time Hi-C techniques have primarily been applied to study the gut and environmental resistome^59–62^.

Despite one clinically relevant strain of *G. vaginalis* (JCM 11026) not containing a plasmid, *G. vaginalis* does engage in horizontal gene transfer, especially within the same subspecies or clade^63,64^. *Gardnerella spp*. can be divided into at least four different clades^64,65^. Within the same clade of *Gardnerella spp*. there is evidence of homologous recombination and between *Gardnerella spp*. clades there is limited (but non-zero) genetic exchange^64,65^. Horizontal gene transfer between different clades appears to be stronger between more closely related clades such as *G. vaginalis* and *G. piotti* than between more distantly related clades^65^. Clade-specific competence genes have been detected in *Gardnerella spp*., but their expression is unclear^65,66^. Thus, it is difficult to draw conclusions about the extent of *Gardnerella spp*.’s uptake of environmental DNA. *Gardnerella spp*. is not the only member of the BV consortium capable of engaging in horizontal gene transfer. Specifically, the TOH-2715 strain of *P. bivia* has two chromosomes and has been shown to harbor multiple antimicrobial resistance genes^67^. One chromosome harbors transposon-encoded resistance to metronidazole (*nimK*) and the other chromosome harbors resistance genes to macrolide and tetracycline (*ermF*, *tet(Q)*) in a structure resembling a transposon, however, the expression of these genes is unknown^67^. In addition to horizontal gene transfer amongst vaginal pathogens, horizontal gene transfer has been reported in *Lactobacillus spp*. and is hypothesized to be attributed to be the cause of *L. crispatus* acquiring sialic acid use genes and fructose and cellobiose transport systems^68^. Horizontal gene transfer has also been reported in *N. gonorrhoeae* (*porB*, *rplB, rplD, rplY*) and *Chlamydia*^69–71^.

The concept of the spread of the resistome is vastly different in yeasts (e.g. *C. albicans*) than in bacteria due to yeast increasing genetic diversity via sexual reproduction^72,73^. At vaginal pH 4, *C. albicans* can switch from a white to opaque phenotype and alter its sexual mating patterns^74^. Additionally, *C. glabrata* has been referred to as a “powerhouse of resistance” and is notable for its widespread -azole resistance in vulvovaginal candidiasis^75,76^. *C. glabrata* undergoes dramatic chromosomal rearrangements, with large chromosomal duplications and translocations that can cause antimicrobial resistance^76^.

### Host factors contributing to the vaginal resistome

Several factors in the vaginal microenvironment can influence the vaginal resistome including vaginal microbial composition, pH conditions, contraceptive use, menstrual cycle, mucus permeability, and mucosal immunology^4^. Multiple distinct vaginal microbiome community state types (microbial composition) have been identified globally in healthy individuals and certain ethnic populations have been shown to be more likely to present with a community state type^4^. Community state types I-III and V and predominantly comprised of *Lactobacilli* (*L. crispatus, L. gasseri, L. iners, L. jensenii*) and are most prevalent in women with European and Asian ancestry^77^. Type IV has an increased anaerobe diversity comprised of *Prevotella, Megasphaera, Sneathia, Gardnerella vaginalis*, and *Fannyhessa vaginae* and is most prevalent in women with African American and Hispanic ancestry^77–79^. However, it is important to note that these studies conducted much of their sampling in North America and Europe and there is a need to diversify sampling to other regions globally to capture different vaginal microbiota compositions^80^. Since several microbes associated with the vaginal microbiome, for example *Gardnerella, Lactobacillus*, and *Prevotella*, can harbor antimicrobial resistance genes, the overall composition of the vaginal microbiome plays a critical role in determining an individual’s susceptibility towards developing resistant infections^81^.

pH conditions of the vaginal microbiome can change based on the disease state. For example, in the absence of infection, the vaginal pH is typically between 3.5–4.5 due to the production of lactic acid by *Lactobacillus spp*. that establishes the acidic environment^82^. Whereas during an infection the vaginal pH rises above 4.5^82^. The growth of microbes in the vaginal microbiome is influenced by pH conditions and certain microbes that can harbor antimicrobial resistance genes preferentially grow under less acidic conditions (e.g. *Gardnerella spp*.)^82^. Therefore, the acidity of the vaginal microenvironment has a direct influence on the population of microbes and is linked with an individual’s susceptibility towards developing resistant infections.

Contraceptive use can also alter the composition of the vaginal microbiome and as a result influence the growth of microbes that harbor antimicrobial resistance genes. Intrauterine contraceptive devices have been shown to alter the vaginal microbiota and contribute to bacterial vaginosis whereas hormonal-based contraceptives do not elicit significant changes to the vaginal microbiota^83^. This difference is likely due to the intrauterine devices directly coming in contact with and disrupting the vaginal microenvironment.

It is also important to consider the effects of the stage of the menstrual cycle on the composition of the vaginal microbiome and the viscosity and permeability of cervicovaginal mucus^84–86^. Gajer et al. have shown that the composition of the vaginal microbiota is highly dynamic over the menstrual cycle and that an individual may experience several different community state type compositions over the duration of their menstrual cycle^84^. Therefore, this suggests that the microbes that harbor antimicrobial resistance genes in the vaginal microbiome may dynamically shift during menstruation. In addition, the viscosity of cervicovaginal mucus typically changes under hormonal control (menstruation) and in pregnancy^85^. When highly viscous, cervicovaginal mucus functions to impede transport of STIs^85^. In the estrogen-dominant follicular phase (pre-ovulation) and ovulation phase of the menstrual cycle, cervical mucus secretion increases and mucus has low friction to allow for easy passage of sperm^87^. At the end of ovulation, the progesterone-dominated luteal low fertility stage begins and the cervical mucus thickens and is more barrier-like^87^. Changes to mucus permeability are often considered in the context of fertility (transport of sperm), however, they are also associated with the transport of STIs. As the viscosity of the mucus increases, transport of STIs is reduced; it is important to note that cervical mucus is not considered a method to prevent STI transmission^88^. Nevertheless, high viscosity cervical mucus provides less opportunity for transport of pathogens harboring antimicrobial resistance genes and therefore mucus permeability and the stage of the menstrual cycle influence an individual’s risk of developing resistant gynecological infections.

**Fig. 1:**
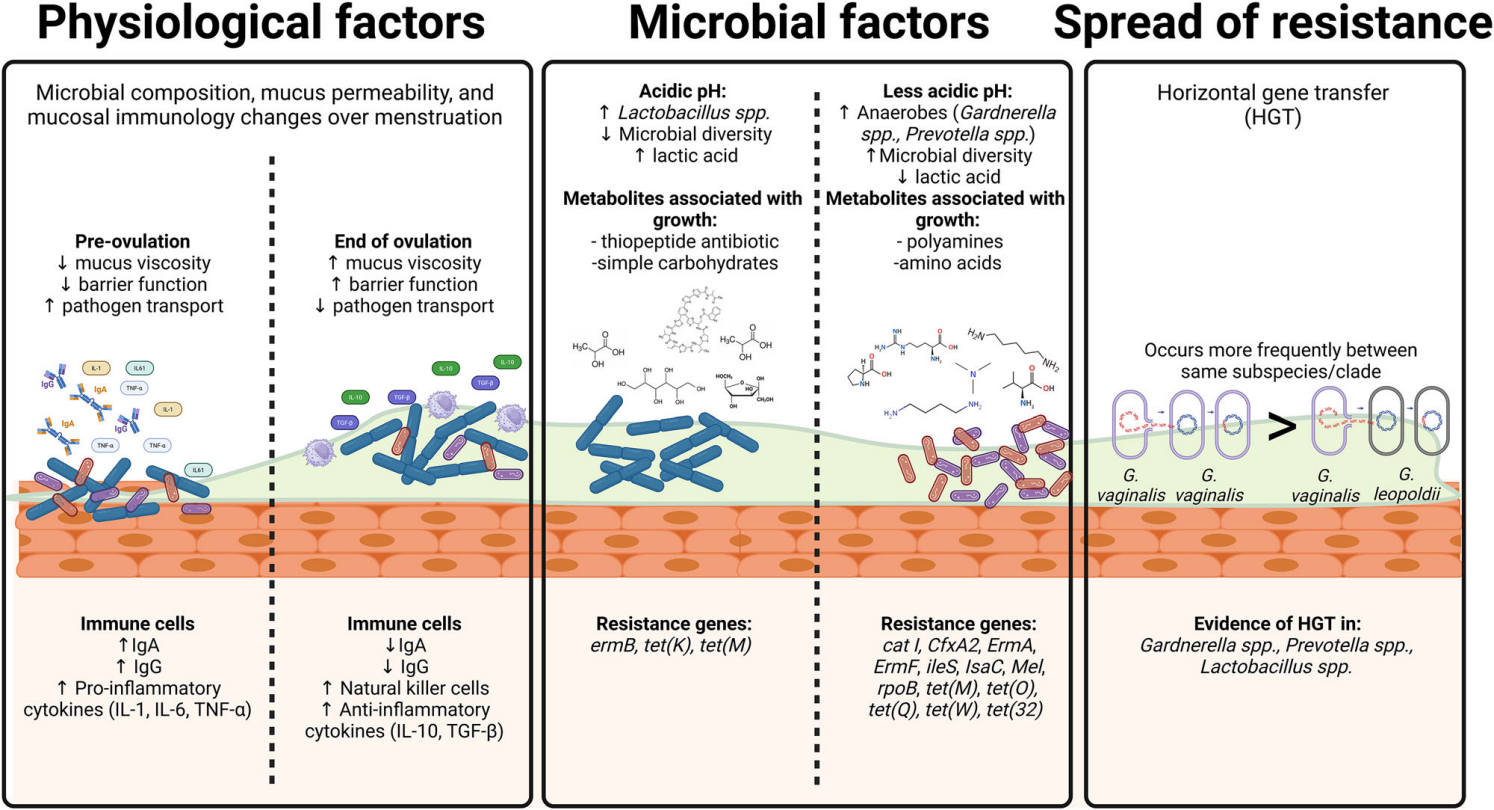
Host physiological and microbial factors that contribute to the spread of antimicrobial resistance genes amongst vaginal BV-associated microbes. Host physiological factors including the phase of the menstruation cycle influence vaginal microbial composition, mucus permeability/viscosity, and mucosal immunology. When mucosal viscosity is low, transport of STIs and microbes harboring resistance genes increases (left). The vaginal microbiota secretes metabolites and small molecules that promote or suppress the growth of microbes harboring antimicrobial resistance genes. *Lactobacillus spp*. secrete lactic acid to maintain an acidic environment and ward off growth of pathogens. Reduction of *Lactobacillus spp*. increases vaginal pH allowing anaerobes to thrive and produce an environment rich in polyamines (middle). Horizontal gene transfer has been shown to occur in BV-associated microbes (*Gardnerella spp., Lactobacillus spp*., and *Prevotella spp*) (right).

In addition to the aforementioned host factors, mucosal immune cells in the vaginal microenvironment influence the inflammatory state by modulating the release of cytokines and chemokines and can shape the vaginal resistome^89^. In a state of infection, such as bacterial vaginosis, mucosal immune cells release pro-inflammatory cytokines (IL-6, IL-8, TNF-α)^89^. In theory the pro-inflammatory cytokines are intended to mitigate infection, but with chronic inflammation the integrity of the cervicovaginal epithelial barrier can be compromised and increase the risk of acquiring STIs and of preterm birth^89,90^. A similar inflammatory state is observed in vulvovaginal candidiasis in which hyphal antigens invoke an immune response driven by pro-inflammatory cytokines such as IL-1β^91^. In most bacterial STIs (chlamydia and gonorrhea), there is a potent inflammatory response that can lead to pelvic inflammatory disease and downstream complications (e.g. ectopic pregnancy, infertility) if untreated^92^. Therefore, patients that present with heightened inflammatory responses may have compromised cervicovaginal epithelial barriers that enable further spread of pathogens and antimicrobial resistance genes and increase their susceptibility towards resistant infections^93^. Figure 1 summarizes how host physiological and microbial factors and horizontal gene transfer contribute to the composition and spread of antimicrobial resistance genes in the vaginal resistome.

## Traditional strategies to combat resistance in the vaginal microbiome

The current first line treatment for BV is antibiotics such as metronidazole or clindamycin^8^. Metronidazole functions to intercept DNA synthesis while clindamycin functions to interrupt protein synthesis^94,95^. Metronidazole is offered in both oral and vaginal suppository formulations; the suppository formulation offers a more targeted therapy but can be cost prohibitive^96^. The first line of treatment for *C. albicans* infection is fluconazole, which functions to inhibit the enzyme that produces ergosterol, a key cholesterol component of the fungal cell membrane^97^. However, fungi, such as *C. glabrata* are becoming increasingly resistant to fluconazole^98^. First line treatment for bacterial STIs includes ceftriaxone (third generation cephalosporin) and azithromycin; however, *N. gonorrhea* has shown resistance to all antibiotics typically prescribed as first line treatments^33^. Specifically, *N. gonorrhea* has shown resistance to beta-lactam, sulfonamide, tetracycline, and macrolide classes of antibiotics^33^. Antimicrobial peptides have also been explored as an antibiotic alternative therapy for treatment of BV infections (Nile tilapia piscidin 4, pexiganan, melittin, cathelicidin-DM) and *C. albicans* biofilms (Scyampcin_44-63_, LL-III, HAL-2) and recurrent vulvovaginal candidiasis (RP504, RP554, RP556, RP557)^99–103^. Further, antimicrobial peptides have been developed to address *N. gonorrhea* (17BIPHE2, LL-37) and *C. trachomatis* (Novispirin G-10, cyto-insectotoxin 1a) infections^104–107^. Alternatively, substances such as pH balancers and natural products (e.g. *Thymbra capitata*) have been evaluated for their activity against vaginal pathogens with mixed results^16,108,109^. Specifically, boric acid (pH balancer) has been used to address resistant *C. glabrata* by interfering with virulence factors, biofilm formation, and hyphal transformation^108,110,111^.

## The microbiome as an emerging therapeutic tool to manage the vaginal resistome

As traditional therapies have failed to meet the increasingly demanding needs of the vaginal resistome, there has been a notable turn to harness the microbiome as a tool to manage and combat resistant vaginal infections. Specifically, vaginally administered probiotics (individual species and consortia) have been widely applied for cases of resistant vaginal infections^112–117^. A clinical trial investigating combinatorial use of intravaginal LACTIN-V (strain of *Lactobacillus crispatus*) with metronidazole resulted in a 30% BV relapse rate 12 weeks post-treatment relative to a 45% relapse rate in the placebo group^112^. When the study was taken out to 24 weeks, both the LACTIN-V with metronidazole and placebo groups had comparable rates of BV relapse^112^. Similarly, another study showed that sustained release of probiotics can reduce recurrence of BV^113^. In this study, 35 women with acute BV were administered vaginal suppositories with either a placebo or with *Lactobacillus fermentum* LF-15 and *Lactobacillus plantarum* LP-01 in a sustained release formulation^113^. Participants used the suppository daily over seven days and eventually at lower frequencies over the span of two months^113^. Following the one week and two month trials, participants that received the probiotic consortia had significantly fewer cases of BV relative to the placebo group^113^. Other combinations of probiotics including *Lactobacillus acidophilus* GLA-14, *Lactobacillus crispatus* CTV-05, *Lactobacillus gasseri, Lactobacillus rhamnosus* HN001, and *Streptococcus thermophilus* have shown various efficacies in reducing recurrent/resistant BV infections^114–117^. Similarly, combinations of probiotics (*Bifidobacterium bifidum*, *Bifidobacterium longum*, *Lactobacillus acidophilus* GLA-14, *Lactobacillus acidophilus* LA02, *Lactobacillus delbrueckii*, *Lactobacillus fermentum* LF10, *Lactobacillus fermentum* LF26, *Lactobacillus helveticus* LA25, *Lactobacillus paracasei* LPC12, *Lactobacillus plantarum* LP115, *Lactobacillus plantarum* P17630, *Lactobacillus rhamnosus* HN001, *Lactobacillus rhamnosus* LRH10) have been explored for the treatment of recurrent vulvovaginal candidiasis with varying levels of success^118–123^. In both BV and vulvovaginal candidiasis, monotherapy with probiotics is often not sufficient to eradicate the infection and prevent recurrence^8^.

While the majority of the aforementioned probiotic studies focused on vaginal administration, it is notable that probiotics have also been administered orally to influence the vaginal microbiome and address recurrent BV and vulvovaginal candidiasis^121,124,125^. The mechanism regarding how the gut microbiome influences the vaginal microbiome has yet to be fully elucidated, however, recent studies have demonstrated a strong association between gut and vaginal microbes^126–128^. In addition to orally and vaginally delivering defined consortia of vaginal microbes, an exploratory study evaluated the efficacy of a vaginal microbiome transplant in women with recurrent and intractable BV^129^. A repetitive vaginal microbiome transplant was required in several participants to prevent recurrent BV, nevertheless, additional larger placebo-controlled studies are necessary to determine the efficacy of vaginal microbiome transplants on addressing resistant vaginal infections^129^.

Alternatively, bacteriophage therapy has been evaluated to treat resistant vaginal microorganisms and biofilms^17,130–132^. Bacteriophages are viruses that are highly targeted for bacteria and several groups have engineered phage endolysins (e.g. PM-477) to eradicate *G. vaginalis* biofilms in BV while leaving *Lactobacillus spp*. intact^17,19,131–133^. Bordigoni et al. isolated a clinical *G. vaginalis* derived phage (vB_Gva_AB1) that may serve as a promising therapy^130^. Bacteriophage therapy has also been explored for treatment of *N. gonorrhea* and *C. trachomatis* infections, yet much more work is needed to validate their clinical efficacy^134,135^. Widespread use of bacteriophage therapy has been hindered by a lack of consensus of dosing frequency, route of administration, proper controls across studies, and potential for developing resistance^136^. Furthermore, there is a lack of widespread infrastructure for direct and timely bench to bedside identification of phage candidates for individual patients.

## Future directions and perspectives for studying and combating the vaginal resistome

Beyond the aforementioned probiotic and bacteriophage approaches to combat resistant gynecological infections, microbial metabolites are a recently identified factor that contribute to the competition and spread of antimicrobial resistance genes amongst microbial communities and are key effectors of genes^137–140^. Microbial metabolism has been leveraged to combat resistant microbes^141–143^ and the metabolites present in a microbiome community may provide a competitive fitness advantage to the microbes that harbor antimicrobial resistance genes^144,145^. For example, carbohydrate and amino acid supplementation has been used to stimulate the tricarboxylic acid (TCA) cycle in antibiotic-resistant gut microbes (*E. coli*, *Edwardsiella tarda*), increasing antibiotic uptake and killing resistant microbes^141–143^. In the context of the vaginal microbiome, when resistant *Gardnerella spp*. is in the presence of a vaginal environment rich in sugars (e.g. maltose, maltotriose, glucose) it proliferates to outcompete other vaginal microbes due to the presence of maltose ABC transporters (MusEFGK_2_I, MalXFGK)^146,147^.

Metabolomics and proteomics have emerged as leading techniques to understand how microbial metabolism contributes to the spread of antimicrobial resistance genes in human and environmental microbial consortia, but have not been widely used to study the vaginal resistome^139^. Both targeted and untargeted metabolomics approaches have been applied to study microbial metabolism in vaginal consortia. Untargeted metabolomics enables wide identification of metabolites in a sample but does not always enable elucidation of the chemical structure^139^. Whereas targeted metabolomics allows for more precise elucidation of the chemical structure of individual metabolites but does not fully capture all metabolites in a sample and relies on the user having previous knowledge of the expected metabolites^139^. Alternatively, proteomics analyses provide a link between structural/functional changes and post-translational modifications to proteins and acquisition of antimicrobial resistance genes^148^. Whole proteome techniques use a “shotgun” approach and are quantitative to determine the abundance of all proteins in a sample^149^. The advantage of whole proteome techniques is that they can provide a robust snapshot of protein expression^149^. An alternative technique is targeted proteomics that quantifies only user-identified proteins of interest^149^. Targeted proteomics enables more precise quantification of selected proteins relative to whole proteome techniques but provides a lower coverage of sample proteins^149^. At present, metabolomics and proteomics studies on vaginal microbes have primarily focused on studying changes in microbial metabolism during infection and have not emphasized changes in the metabolism of resistant vaginal microbes. Nevertheless, here we summarize key findings of metabolites associated with gynecological infections and provide prospective on how these tools may be leveraged in the future to study resistant vaginal microbes.

Zhu et al. and Bloom et al. used a combination of targeted metabolomics and lipidomics to study the metabolism of *L. iners*, a microbe associated with recurrent cases of BV^150,151^. While *L. iners* has been shown to not interfere with *G. vaginalis*, individuals colonized by *L. iners* present with an increased risk of developing BV^152^. Zhu et al. found that long chain fatty acids (oleic acid, linoleic acid, palmitoleic acid) inhibited the growth of *L. iners* while promoting the growth of *L. crispatus*^150^. Conversely, Bloom et al. determined that *L. iners* growth is dependent on L-cysteine^151^. Other techniques including targeted and untargeted metabolomics and multi-omics techniques have been used to identify metabolites associated with a range of bacterial vaginosis and cervicovaginal pathogens that can harbor antimicrobial resistance genes. Specifically, Srinivasan et al. performed a targeted metabolomics study on vaginal fluid from individuals with and without symptomatic bacterial vaginosis and found strong positive associations between the abundance of putrescine, cadaverine, trimethylamine, succinate, tyramine, and deoxycarnitine and negative associations between the abundance of maltose, maltotriose, maltohexose, arachidonate, carnitine, lactate, urea, and reduced glutathione and BV pathogens (*G. vaginalis, P. bivia*, and *F. vaginae*)^153^. Zuend et al. also used targeted metabolomics to identify metabolites associated with the growth of *Prevotella* (xanthine, hexose-phosphate, hexose, *N-*acetyl alanine, 12-hydroxyeicosatetraenoic acid, 13-hydroxyoctadecadienoic acid) and *Lactobacillus spp*. (homovanilate, lactate, adenosine, succinate, phenyllactate) in cervicovaginal inflammation^89^. Alternatively, Bokulich et al. used untargeted metabolomics to identify metabolites associated with the growth of BV pathogens (pipecolate, *N*-acetyl-cadaverine, deoxycarnitine) and *L. crispatus* (*N*-acetyl methionine sulfoxide)^154^. Oliver et al. and Challa et al. used multi-omics techniques to identify positive associations between metabolites and *L. crispatus* (mannitol, fructose, indole-3-lactate) and BV pathogens (beta-leucine, methylimidazole, acetaldehyde, dimethylethanolamine, L-arginine, beta cortol, succinic acid, malic acid, eicosenoic acid, carnitine, proline, valine)^155,156^. Metatranscriptomics and high performance liquid chromatography (HPLC) analyses have enabled identification of vaginal derived metabolites from *L. gasseri* (lactocillin) and *Dermabacter vaginalis* (dermazolium A) that exhibit antimicrobial activity against *G. vaginalis* and MRSA^157,158^. Finally, Bonnardel et al. used a proteome approach to predict carbohydrate-binding proteins that correlated to the pathogenicity of vaginal microbes^159^. Identification of metabolites that promote or suppress the growth of microbes that carry antimicrobial resistance genes can assist in the development of new therapies to target resistant vaginal microbes. Table 2 provides a summary of metabolites that are associated with the growth of vaginal microbes that have been shown to harbor antimicrobial resistance genes.

**Table 2:** Relationships between microbial metabolites and growth of microbes that can harbor antimicrobial resistance genes in the vaginal microbiome

Metabolite or Metabolic pathway	Microbe of origin	Effect on growth of vaginal microbes that can harbor ARGs	Key technique(s)	Reference
Maltose, Maltotriose, Maltohexose, Arachidonate, Carnitine, Lactate, Urea, Reduced glutathione	**--**	**↓***G. vaginalis* ↓ *P. bivia* ↓ *F. vaginae*	Targeted metabolomics	Srinivasan et al. ^153^.
Oleic acid, Linoleic acid, Palmitoleic acid	**--**	**↓***L. iners*	Untargeted lipidomics	Zhu et al. ^150^.
Hexose, Pyruvate, Lactate	**--**	**↓***Lachnospiraceae* ↓ *S24-7 group*	16S rRNA sequencing	Brownlie et al. ^177^.
Lactocillin	*L. gasseri*	**↓***G. vaginalis* ↓ *E. faecalis* ↓ *S. aureus*	Meta-transcriptomics	Donia et al. ^157^.
Dermazolium A	*Dermabacter vaginalis*	**↓**MRSA ↓*B. epidermidis*	HPLC	Kim et al. ^158^.
Lactic acid, Nicotinamide, Nicotinate	*--*	**↓***C. albicans*	Ultra-high-performance liquid chromatography coupled with Q-Exactive Orbitrap mass spectrometry	Zhao et al. ^178^.
Tartaric acid	*--*	**↑***G. vaginalis* ↑ *F. vaginae*	16S rRNA sequencing, LC-MS metabolomics	Challa et al. ^156^.
Putrescine, Cadaverine, Trimethylamine, Succinate, Tyramine, Deoxycarnitine	*--*	**↑***G. vaginalis* ↑ *P. bivia* ↑ *F. vaginae*	Targeted metabolomics	Srinivasan et al. ^153^.
Xanthine, Hexose-phosphate, Hexose, *N*-acetyl alanine, 12-hydroxy-eicosatetraenoic acid, 13-hydroxy-octadecadienoic acid	*--*	**↑***Prevotella*	Targeted metabolomics, proteomics	Zuend et al. ^89^.
Homovanilate, Lactate, Adenosine, Succinate, Phenyllactate	*--*	**↑***Lactobacillus spp*.	Targeted metabolomics, proteomics	Zuend et al. ^89^.
L-cysteine	*--*	**↑***L. iners*	Shotgun metagenomics, Targeted metabolomics	Bloom et al. ^151^.
Mannitol, Fructose, Indole-3-lactate	*--*	**↑***L. crispatus*	Shotgun metagenomics, Lipidomics	Oliver et al. ^155^.
Pipecolate, *N*-acetyl-cadaverine, Deoxycarnitine	*--*	**↑***Prevotella*, *↑Streptococcus*, *↑Megasphaera*, ↑ *F. vaginae*, ↑ *S. amnii*	Untargeted metabolomics	Bokulich et al. ^154^.
*N-*acetyl methionine sulfoxide	*--*	**↑***L. crispatus*	Untargeted metabolomics	Bokulich et al. ^154^.
Beta-leucine, Methylimidazole, Acetaldehyde, Dimethyl-ethanolamine, L-arginine, Beta cortol, Succinic acid, Malic acid, Eicosenoic acid, Carnitine, Proline, Valine	*--*	**↑***G. vaginalis* ↑ *F. vaginae*	16S rRNA sequencing, LC-MS metabolomics	Challa et al. ^156^.
D-arabitol, Palmitic acid, Adenosine	*--*	**↑***C. albicans*	Ultra-high-performance liquid chromatography coupled with Q-Exactive Orbitrap mass spectrometry	Zhao et al. ^178^.

Beyond metabolomics and proteomics techniques to study microbial metabolism, Raman spectroscopy approaches have recently been used to study antimicrobial resistance^160–168^. Raman spectroscopy identifies molecules based on their unique vibrations and light scattering abilities, thus leading to separate fingerprints for individual molecules and metabolites^169^. A single microbe’s Raman fingerprint is composed of the metabolites it houses including DNA, RNA, lipids, and proteins. Therefore, if a single microbe’s metabolism changes, such as in antimicrobial resistance, then Raman spectroscopy can be used to detect the deviation from the baseline fingerprint. Raman spectroscopy is particularly advantageous because it enables rapid culture-free analysis of microbial metabolism^160^. Further, Raman spectroscopy can enable identification of “microbial dark matter,” or previously uncultivated microbes, and sample measurement is non-invasive allowing for re-use in other experiments^170,171^. Gram-positive and gram-negative bacteria can readily be discerned using Raman spectroscopy due to large differences in molecular structure^169^. Additionally, Raman has been applied to differentiate between planktonic and sessile (biofilm) growth states of microbes due to unique metabolomic shifts that occur when microbes are in a biofilm^172^. Single-cell Raman radioisotope techniques have been coupled with targeted metagenomics to study the resistome in soil samples^173^. Currently, Raman has primarily been applied to study resistance in gut and environmental microbes, but has recently been applied to identify fingerprints for common vaginal microbes and fungi including *Lactobacillus spp., Bifidobacterium spp., G. vaginalis, P. bivia, F. vaginae, C. albicans, C. glabrata*, and *Trichomonas vaginalis*^174^. Raman has also been used to distinguish *Chlamydia trachomatis* and *N. gonorrhea* metabolites for rapid screening^175^. Specifically, infectious elementary bodies of *C. trachomatis* were characterized by the presence of unique cell surface proteins whereas *N. gonorrhea* was characterized by the presence of adenine, guanine, and surface co-enzyme nicotinamide adenine dinucleotide^175^. By identifying the metabolic fingerprint of resistant vaginal microbes, Raman spectroscopy may serve as a useful tool to uncover the dynamics of resistant microbes in the vaginal resistome.

While metabolomics/proteomics and Raman spectroscopy techniques have primarily been used to study changes in microbial metabolism in resistant gut and environmental microbes and resistomes, these technologies offer the potential to provide critical insight into the metabolic shifts that occur in resistant microbes in the vaginal resistome. By understanding the metabolic changes in resistant vaginal microbes, new therapies can be developed that target key metabolic pathways. Table 3 highlights the advantages and limitations of emerging technologies that can be applied to study microbial metabolism and spread of antimicrobial resistance genes in the vaginal resistome.

**Table 3:** Advantages and limitations of emerging technologies that offer potential to study the vaginal resistome

Technique	Advantages	Limitations	References
***Techniques related to studying the microbial metabolism of resistant microbes***			
Untargeted metabolomics Targeted metabolomics	-Broad identification of metabolites in sample -More precise determination of chemical structure of individual metabolites	-Does not enable determination of chemical structure -Does not fully capture all metabolites in sample -Relies on user having prior knowledge of expected metabolites	Bokulich et al. ^154^. Srinivasan et al. ^153^., Zuend et al. ^89^., Bloom et al. ^151^.
Whole proteomics Targeted proteomics	-Quantitative to determine abundance of all proteins in sample -Robust snapshot of protein coverage and expression -More precise quantification of selected proteins	-Less precise quantification of select proteins -Lower coverage of sample proteins	Zuend et al. ^89^.
Raman spectroscopy	-Rapid culture-free analysis -Identify previously uncultivated microbes -Sample measurement non-invasive	-Raman spectra can vary based on growth stage of individual microbe -Lack of standardization of technique	Singh et al. ^160^., Ma et al. ^161^., Pavlicek et al. ^162^., Yang et al. ^164^., Germond et al. ^165^., Thomsen et al. ^167^.
***Techniques related to studying the spread of antimicrobial resistance genes in the resistome***			
Hi-C metagenomics Meta3C metagenomics Long-read metagenomics	-Culture-free -Trace mobile genetic elements to microbial origin -Culture-free -Trace mobile genetic elements to microbial origin -Lower burden of sample preparation -Can improve accuracy of linking antimicrobial resistance genes to microbial origin	-Noisy signal if repetitive sequences not removed -Requires biotin-label -Require deep sequencing to link mobile genetic elements to microbes -Complex sample preparation -Costly -Extracting high-quality long DNA fragments	Kent et al. ^59^., Kalmar et al. ^60^., McCallum et al. ^61^., Yaffe et al. ^62^., Tams et al. ^179^. Bickhart et al. ^180^., van der Helm et al. ^181^.
Metatranscriptomics	-Provides information on active expression of individual resistance genes	-Costly -Large amount of RNA required	Pinheiro et al. ^182^., de Nies et al. ^183^., Marcelino et al. ^184^.
Multi-omics	-Provide snapshot of discriminating molecular features to distinguish antimicrobial resistance phenotypes -Link microbial resistance genes to metabolites and gene expression	-Computationally intensive and complex -Costly -Missing samples across analyses -Lack of consensus in data integration technique	Rohart et al. ^185^., Ceccarani et al. ^186^., France et al. ^187^.

In summary, there is a pressing need to understand the complex environment of the vaginal microbiota that allows resistance to develop and to design novel therapies that are less prone to developing resistance. Vaginal microbes associated with bacterial vaginosis, vulvovaginal candidiasis, *N. gonorrhoeae*, and *Chlamydia* can harbor antimicrobial resistance genes and contribute to the vaginal resistome. Antibiotic and microbiome-based approaches (e.g. probiotic, bacteriophage) have been used to combat resistant gynecological infections with varying degrees of success. While there are a range of innovative computational tools (Table 3) at the disposal of researchers to track the spread of antimicrobial resistance genes in gynecological infections and develop novel therapeutics, many challenges remain including optimizing the techniques to handle the relatively low biomass samples of the vaginal microbiome and standardizing microbiome analyses^5,176^. Ultimately, the infrastructure to study the spread of antimicrobial resistance genes and to develop therapies exists but it has primarily focused on gastrointestinal applications rather than gynecological. Therefore, to move the field forward it is necessary to emphasize translation and optimization of existing tools for gynecological infections.

## Data Availability

No datasets were generated or analysed during the current study.
